# Artificial Intelligence, Important Assistant of Scientists and Physicians

**DOI:** 10.31661/gmj.v9i0.2048

**Published:** 2020-11-11

**Authors:** Aliasghar Karimi, Hamed HaddadPajouh

**Affiliations:** ^1^Noncommunicable Disease Research Center, Fasa University of Medical Science, Fasa, Iran; ^2^Cyber Science Lab, University of Guelph, Guelph, Ontario, Canada

**Keywords:** Artificial Intelligence, Noncommunicable Disease, Machine Learning

The human mind has several obstacles and limitations to remember and apply the thousands of medical information learned at medical school quickly. On the other hand, knowledge of medicine is proliferating. Hence, the analysis of the hundreds of papers, journals, and textbooks are impossible for a clinician. However, in evidence-based medical practice, physicians must be used recent guidelines and papers. Due to a report, most diagnostic errors in medical care are related to the wrong cognitive by health care workers [1,2]. Also, medical errors are one of the significant causes of death in the United States that most related to human errors [3,4]. Since the 1950s, physicians and computer scientists have tried to use the capacity of computers as a decision support system to facilitate clinical decisions [5]. Artificial intelligence (AI) is a branch of computer sciences rapidly being adopted in medicine and many other fields. AI technology tries to mimic human behaviors and cognitive functions to be learning and solve a problem similar to a human [6]. Since AI uses computer capabilities in processing and memory management, it does not have human limitations to analyze and interpret millions of information about disorders in less than a minute. In 1976, for the first usage of AI in medicine, Gunn et al. used this technology to diagnose acute abdominal pain [7]. the usage of AI in medicine was in medical algorithms to help physicians diagnose a disease between different diagnoses. Today, AI agents are significantly developed in different environments. The common AI technology is image processing and computer vision that are useful for analyzing and classifying the radiologic or microscopic images. Therefore, AI assists the measurements and diagnosis could be more convenient and faster for radiologists and pathologists. The other AI technologies are Artificial neural networks (ANN), Machine learning, Convolutional neural network (CNN), and Deep learning that are useful to other medical specialists [8]. on the other hand, the AI has a significant role in preventing and managing chronic diseases such as diabetes mellitus [9]. Furthermore, developing smart gadgets and wearable devices that use AI has a beneficial impact on preventing, diagnosing, predicting prognosis, and managing non-communicable diseases [10]. The usage of this technology is growing up every day; however, there are some ethical concerns about apply AI in medicine [11]. on the other hand, there is not enough evidence about the proficiency of AI in medical practice. So, several high-quality studies must be done to understand the pros and cons of AI usage in medicine. Also, several websites and software work with AI to help clinicians and researchers interpret and analyze the papers. However, the AI could be more useful to scientific writing and data extraction from numerous articles. It seems that in this decade, AI will be an undeniable assist of scientists and physicians, and more evidence must be published in this area.
